# Petroleum Carcinogenicity and Aerodigestive Tract: In Context of Developing Nations

**DOI:** 10.7759/cureus.1202

**Published:** 2017-04-30

**Authors:** Sunali Khanna, Amit S Gharpure

**Affiliations:** 1 Municipal Corporation of Greater Mumbai, Nair Hospital Dental College; 2 Private Practice, Mumbai

**Keywords:** aerodigestive tract, petroleum exposure, carcinoma, environment, environment health, air pollution, cancer, environment and public health

## Abstract

Head and neck cancers from a diverse group of neoplasms, the occurrence of which can be attributed to habitual tobacco use, race, alcohol consumption, ultraviolet (UV) exposure, occupational exposure, viruses, and diet. The surging incidence rates reflect the prevalence of risk factors such as tobacco use (smoked and smokeless), betel nut chewing, urbanization and issues relating to urban air quality. Urbanization and development have catalyzed a multifold rise in levels of pollution in metropolitan cities. Ever-increasing consumption of fuels to meet demands of the growing population coupled with industrial activity has adversely affected the air quality, especially in developing countries. The cause most neglected in risk assessment of aerodigestive tract cancer research is that from petroleum exposure. The global issue of petroleum carcinogenicity has assumed high proportions. Polycyclic aromatic hydrocarbons and heavy metals are essential constituents of total petroleum hydrocarbons which infiltrate into the environment and are recognized worldwide as priority pollutants because of their toxicity and carcinogenicity. High levels of sulfur dioxide, nitrogen dioxide, ozone, carbon monoxide, ammonia and particulate matter PM_10  _has skyrocketed aerodigestive tract diseases especially carcinomas. The identification of specific biomarkers and role of metal ions in aerodigestive tract cancers will indicate the molecular basis of disease to provide quality care for patients confronting new threats from climate-sensitive pathologies. There is an urgent need to evaluate existing public health infrastructure so as to take ameliorative and adaptive measures.

## Introduction and background

The environment has always had a far-reaching effect on public health. Rapid economic development, population growth, and urbanization in developing countries have resulted in high levels of air pollution. Poor air quality caused by anthropologic actives in the 20th century has led to the rise of numerous diseases. There have been several studies and reviews which assessed the impact of air pollution on general health [1-3]. On account of the alarming rise of diseases caused by pollution, there is a need to focus government efforts and research on reducing air pollution levels, promotion use of clean energy sources and investigate the harmful effects of air pollution on humans.

Cancers of the aero-digestive tract are a grave problem showing an upward trend in various parts of the world. They constitute approximately four percent of all malignancies and include cancer of the lip, tongue, major salivary glands, gums and adjacent oral cavity tissues, the floor of the mouth, tonsils, oropharynx, nasopharynx, hypopharynx and other oral regions, nasal cavity, accessory sinuses, middle ear, and larynx [4]. A majority of laryngeal, oropharyngeal and hypopharyngeal cancers are caused by tobacco and alcohol [4]. Although tobacco consumption and alcohol have been attributed as the primary cause of such cancer, high rates of nasal adenocarcinoma have also been recorded in people who work in the furniture and cabinetmaking industries, boot and shoe manufacture, early processes for the manufacture of isopropyl alcohol, and nickel refining [4].

There is a growing body of evidence which has linked the air pollution to cancer of the aerodigestive tract [5-6], an association which is supported by World Health Organisation (WHO) [7]. Outdoor air pollution and particulate matter have been officially classified as a group one carcinogen by The International Agency for Research on Cancer (IARC), a WHO cancer agency [7]. This has had a far-reaching effect on the acknowledgment of the detrimental impact of air pollution, a leading environmental factor, in cancer deaths. Thus, the association between pollutants from industrial products and aero-digestive tract cancers needs to be thoroughly evaluated and stringent measures need to be employed to curb the ill-effects of such products released into the environment.

## Review

### Aerodigestive tract cancer

Having more than 2000 new cases reported globally each year, oral cancer has emerged as a significant public health concern [8]. In South Central Asia, 80% of the head and neck cancers are found in the oral cavity and oropharynx [2]. Most oral malignancies begin as leukoplakia, erythroplakia, and erythroleukoplakia which are inflammatory lesions [9-10]. Various etiologic factors have been recognized as causative agents of cancerous lesions of the oral cavity such as tobacco use, frequent alcohol consumption, a compromised immune system, the use of areca nut, previous history of cancer, dietary habits and other factors such as infection with certain types of human papillomaviruses [11-12]. Additionally, areca nut and betel quid chewing have also known to cause oral precancerous lesions such as oral leukoplakia and oral submucous fibrosis [13]. Rapid urbanization observed in developing countries has led to an unhealthy lifestyle due to an increased access to various forms of tobacco causing a rise in the incidence of oral cancer [2]. Lack of awareness on oral health, hygiene and available treatment options in urban populations of developing nations [14-16] further compounds the problem.

Other than the association between cancer and smoking and alcohol consumption, there have been several studies in the past which were undertaken to find out the incidence of cancer specifically caused by harmful products from polluted air. A multi-centre urban study consisting of 8111 subjects showed that air pollution consisting of fine particulates, including sulfates was positively associated with lung cancer mortality [17]. Another study with a 15-year follow-up consisting of 6,338 non-smoking adults showed an association between increased risk of developing lung cancer and air pollution consisting of sulfur dioxide, ozone, and particulate matter 10 (PM10) [18]. In Asian countries, a sharp increase in lung cancer has been noted, especially over the last three decades. A study conducted in China has demonstrated an increase in lung cancer cases and the incidence is much higher in urban populations as compared to rural populations [19]. Such high levels of cancer in urban regions can be attributed to the greater amounts of pollution in these locations as compared to rural areas. Similar results have been demonstrated in India, where urbanization and industrialization are happening at a rapid rate [6]. Higher incidence of lung cancer is being noted in non-smoking women in India and air pollution is a likely factor for these findings [6]. Additionally, there has been a rise in other cancers of the head and neck regions such as oral, laryngeal, and pharyngeal cancers likely caused due to harmful compounds in the polluted air [20-21]. In general, the incidence of head and neck cancers in the Indian male population is showing an upward trend [6]. Thus, air pollution is imposing a serious health burden in the Asian countries.

### Air pollution and petroleum products

The rise of urbanization has seen the increase in industrial activities and number of motor vehicles all of which generate pollutants into the atmosphere. The release of volatile organic compounds into the environment due to industrial activities and use of petroleum based products has contributed significantly to the urban air pollution. Petroleum and diesel products released from car exhausts contain carcinogenic products such as polycyclic aromatic hydrocarbons (PAHs), alkylated PAHs, alkylbenzenes and alkanoic acids [22]. Gasoline contains large numbers of dangerous and cancer-causing chemicals such as benzene, butadiene, toluene, ethylbenzene, xylene, trimethylpentane, methyl tert-butyl ether (MTBE) and many others which have shown evidence of increased risk of leukemia, kidney, liver, brain, pancreas, aerodigestive tract and prostate cancers [23]. Compounds such as benzene, ethylbenzene, xylenes, 1,1,1-trichloroethane, trichloroethylene, tetrachloroethylene, carbon tetrachloride, chloroform, which are widely released from industries into the environment are known to be toxic and/or carcinogenic substances [24-25]. The harmful effects of increasing ambient concentrations of pollutants are dangerous for public health, more so, in densely populated urban areas [26]. Atmospheric photochemistry converts the more reactive volatile compounds by oxygenation and through reactions with oxides of nitrogen to secondary volatile organic compounds, free radicals, ozone, and subsequently to smog organic aerosols, all of which are detrimental to public health [25-27]. One of the major components of petroleum products is polycyclic aromatic hydrocarbons (PAHs) which are highly condensed aromatic compounds. Common natural sources of PAHs mainly include forest fires, extrusive volcanism, and depositional transformation of biogenic precursors [28]. High demand of fossil fuels have caused and increased release of PAHs into the urban environment due to various human activities such as vehicle emissions, coal and fossil fuel combustion, petroleum refining, straw and firewood burning, industrial processing, chemical manufacturing, oil spills, and coal tar [29]. The PAHs produced by such human activities have been detected in the atmosphere, precipitation, urban surface dust, sediment, and soil, and they have likely caused long-term significant damage to the human respiratory system [30]. They also pose an increased risk for mutagenic and carcinogenic diseases in the urban population [30].

Other than rising in industrialization, there has been a significant increase in the use of indoor fuels such as coal and wood. An increased incidence of aerodigestive tract cancers, especially hypopharyngeal cancer and lung cancer, has been linked to increased lifetime exposure to such fuels [31]. High levels of aerodigestive cancers have been reported in China and Brazil, where the air pollution caused by indoor fuels is on the rise [32-33]. The increased incidence of such cancers in the Indian population could also be a reflection of air pollution caused by a rise in the use of these combustible domestic fuels. Thus, air pollution caused by the use of petroleum products has had detrimental effects on public health and such products likely play a role in the higher incidence of aerodigestive tract cancers.

### Mechanism of action of petroleum pollutants

**Figure 1:**
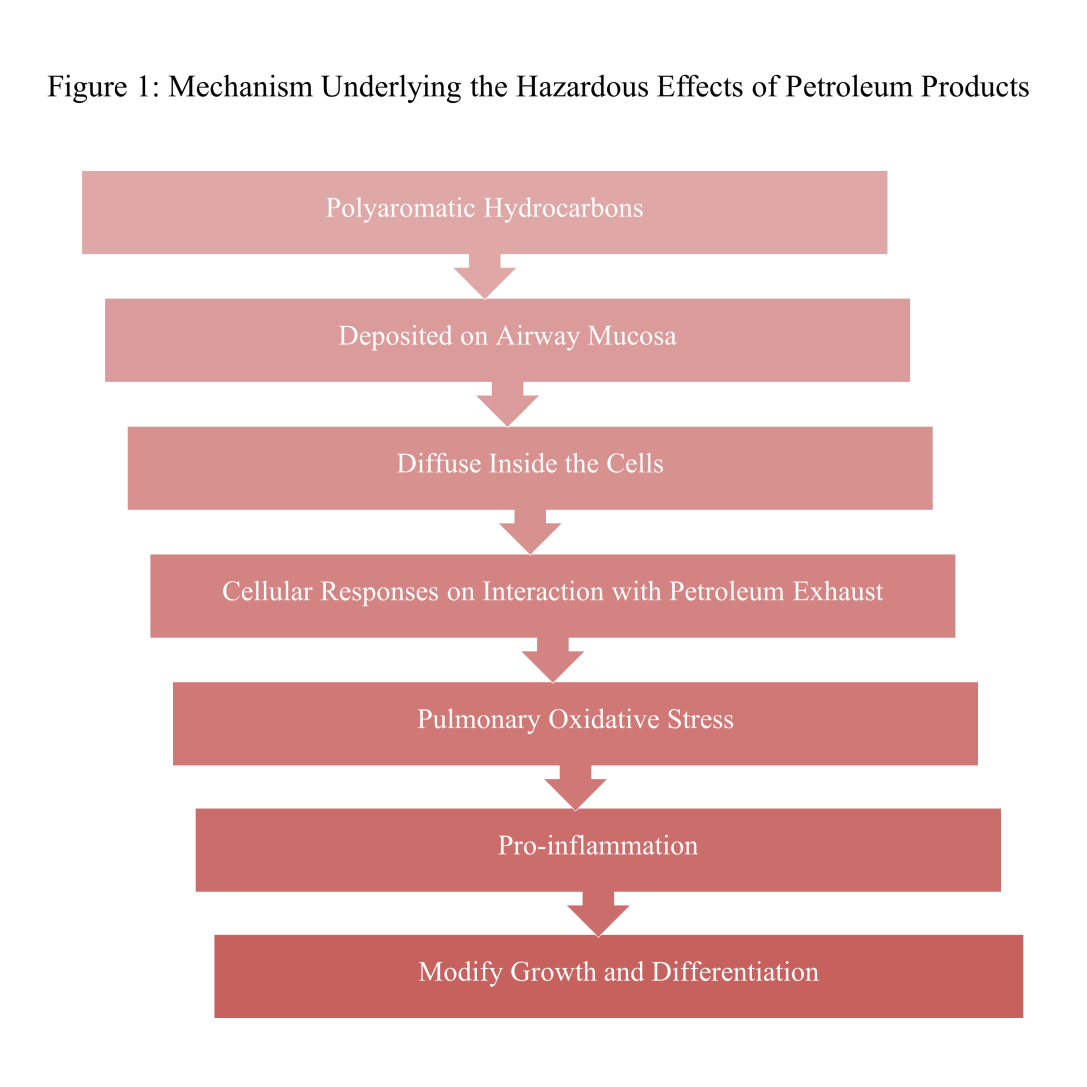
Mechanism underlying the hazardous effects of petroleum products

Although the association between smoking and cancers of the aerodigestive tract is well established, there have been few articles which have directly studied the effects of air pollutants on cause and incidence of aerodigestive tract cancers. The complexity of the nature of aerodigestive tract cancers and the variety of compounds present in the pollutants make it difficult to verify the exact mechanism of the cause of such diseases. However, there have been several studies in which, have shown an association between cancer mortality rates and air pollution [34-35]. The possible mechanism of action can be seen in (Figure 1).

The various compounds from pollutants are inhaled and get deposited on the airway mucosa. From the mucosa, they diffuse into the cells and modify cell growth and differentiation. These molecules thus evoke a cellular response and result in pulmonary oxidative stress. The pollutants cause pro-inflammatory response from the tissue and ultimately result in genotoxicity.

Recently, two animal research papers studied the effect of air pollution on mice [36]. In the first paper [36], laboratory mice were divided into two groups, one group had the mice kept in a controlled polluted environment in an industrial area. The mice from the second group were kept in a rural location away from the first group. Both the groups were then exposed to the ambient air in their respective locations for a short period and were allowed to breed under the same conditions in a standardized animal care facility. On examination of the progeny of the experimental animals, it was found that the rate of mutation in deoxyribonucleic acid DNA was almost two times higher in the progeny exposed to polluted air as compared to the ones of the rural control group. Such high levels of DNA mutation was likely caused by the exposure to polluted air [36]. In the second study [37], the mice were kept in polluted urban locations, with one subgroup exposed to the ambient air and the other subgroup kept in a clean air system maintained with high-efficiency particulate filters. The mice were then bred and the DNA mutation rates of their progeny were compared. The mutation rates were the highest in progeny exposed to polluted ambient air whereas filtered air in the same environment reduced the paternal mutation rate by nearly 52% [37]. A strong association was seen between the concentration of polycyclic aromatic hydrocarbons (PAHs), suspended particulate in polluted air and the rate of the mutation [37]. Thus, both these studies clearly demonstrated how hazardous chemicals from the polluted air can cause DNA mutation.

Pollutants in the air can also lead to DNA damage and protein adducts. The particulate matter in the air may induce inflammation and oxidative stress and over a long period of time, may lead to aerodigestive tract cancers [38-39]. Long-term DNA or protein damage was reported in populations in polluted areas and was linked to cancer [40]. Additionally, increased levels of detected DNA adducts were seen in healthy, non-smoking subjects and these subjects eventually developed lung cancer [41-42]. Thus, high levels of DNA and protein damage were seen in the populations in polluted areas and could be indicative of higher chances of developing aerodigestive tract cancers.

Various DNA and protein markers are being explored to reflect the effects of air pollution in humans. A recent study has shown plasma levels of p53 and p21 proteins to be associated with environmental exposure to benzopyrene and carcinogenic PAHs [43]. Also, trace elements such as copper, zinc, selenium and molybdenum have been used as markers for oral submucous fibrosis and oral squamous cell carcinoma and could potentially act as indicators for oral cancer lesions [44-46]. All these publications have paved way for further studies which need to be conducted to examine the penetrating effects of air pollution on our genome and proteins that cause cancer.

## Conclusions

Although the exact mechanism of action of air pollutants on aerodigestive tract cancer has yet to be understood in detail, there is sufficient evidence to show a relation between them. Whether such cancers are etiologically or genetically similar to those caused by cigarette smoking needs to be examined. The majority of diseases are the consequence of both environmental exposures and genetic factors and a better understanding of genetic influences on environmental response could lead to more accurate estimates of disease risks and provide a basis for disease prevention and early intervention programs. Efforts need to be undertaken, not only to conduct studies on the biology of air pollution associated cancers, but also towards promotion of clean energy, reduction of air pollution, and the establishment of effective policies to regulate the release of polluted air into the atmosphere. Such efforts once carried out successfully, would significantly reduce cancer incidence and the related health burdens in developing countries worldwide.
